# Explainable machine learning for 2-year functional outcomes in older adults with aneurysmal subarachnoid hemorrhage in North China: external validation and health-system context

**DOI:** 10.3389/fneur.2026.1955260

**Published:** 2026-09-25

**Authors:** Wengao Zhang, Gang Li, Xirui Wang, Yue Gao, Pengfei Gao, Xinyu Yang, Chenglu Lu

**Affiliations:** 1 Department of Neurosurgery, Cangzhou Central Hospital, Cangzhou, Hebei, China; 2 Department of Neurosurgery, Tianjin Medical University General Hospital, Tianjin, China; 3 Department of Pathology, Tangshan People's Hospital, Tangshan, Hebei, China

**Keywords:** aneurysmal subarachnoid hemorrhage, external validation, hospital capacity, machine learning, older adults, public health, SHAP

## Abstract

**Background:**

Older adults with aneurysmal subarachnoid hemorrhage (aSAH) have heterogeneous long-term outcomes, and differences in treatment pathways and hospital capacity may complicate early risk stratification.

**Methods:**

This multicenter cohort study included adults aged ≥60 years with aSAH in North China. Of 1,132 admissions at four hospitals from January 2017 to September 2023, 914 patients with 2-year follow-up were divided 7:3 into training (*n* = 640) and internal-validation (*n* = 274) cohorts. An independent cohort screened 602 admissions from January 2017 to January 2021 and included 499 patients. Poor outcome was defined as modified Rankin Scale 3–6 at 2 years. Least absolute shrinkage and selection operator regression with 10-fold cross-validation selected predictors, and 10 machine-learning algorithms were compared. Discrimination, calibration, decision-curve analysis, and SHapley Additive exPlanations (SHAP) were evaluated.

**Results:**

Among 1,413 patients, 583 (41.3%) had a poor 2-year outcome. Gradient boosting machine (GBM) AUCs were 0.863 (95% CI, 0.835–0.891), 0.834 (0.785–0.883), and 0.791 (0.749–0.833) in the training, internal-validation, and external-validation cohorts, respectively. External-validation accuracy was 0.752, sensitivity 0.661, specificity 0.808, precision 0.683, and F1 score 0.672. GBM had the highest external AUC point estimate but did not lead every classification metric. SHAP ranked Hunt–Hess grade, age, World Federation of Neurosurgical Societies grade, and treatment as the most influential features. Unadjusted survival differed across treatment and hospital-capacity strata (both log-rank *p* < 0.0001).

**Conclusion:**

An explainable GBM retained moderate discrimination during external validation and may support admission-stage risk stratification in older adults with aSAH. The health-system findings are associative, and prospective recalibration, fairness assessment, and impact evaluation are required before clinical or public-health deployment.

## 1 Introduction

Aneurysmal subarachnoid hemorrhage (aSAH) is a neurological emergency with substantial early mortality and persistent disability among survivors (1, 2). Although aSAH occurs at all adult ages, population aging is increasing the number of older patients for whom frailty, comorbidity, physiological reserve, treatment selection, and access to specialized neurovascular care must be considered simultaneously. Chinese studies have also shown clinically important regional and treatment-related heterogeneity (3, 4). For patients and families, the question is not limited to surviving the index hospitalization; the probability of meaningful function months to years later is central to shared decision-making and rehabilitation planning.

Existing outcome scores and prognostic models commonly emphasize age, neurological grade, imaging findings, and acute complications (5–7). Machine-learning approaches may capture nonlinearities and interactions that conventional regression models miss, and several models have shown promising discrimination in aSAH (8–10). However, apparent performance can be inflated by overfitting, single-center sampling, data leakage, or *post hoc* model selection. External validation in a clinically different population is therefore essential, and point estimates should be interpreted together with calibration, clinical utility, and transportability (11). Explainability is equally important because risk estimates that cannot be connected to recognizable clinical or system-level information are difficult to audit and implement.

Health-system context is particularly relevant in time-sensitive neurovascular disease. Higher institutional case volume and specialized capacity have been associated with aSAH outcomes, although these relationships are affected by referral patterns and case mix (12, 13). In North China, differences in hospital capability, transfer pathways, and access to microsurgical or endovascular treatment may influence both the data distribution encountered by a prediction model and the downstream actions available after a high-risk prediction. A useful digital-health tool must therefore be externally valid, transparent, and framed as decision support rather than a substitute for clinical judgment or a basis for withholding treatment.

We developed and externally validated machine-learning models for poor 2-year functional outcome among adults aged ≥60 years with aSAH in North China. We compared 10 algorithms, interpreted the gradient boosting machine (GBM) model using SHapley Additive exPlanations (SHAP), and explored survival patterns by treatment modality and hospital-capacity category. We hypothesized that an explainable model based on information available during the index admission would retain discrimination in an external cohort and reveal clinically and health-system-relevant contributors to long-term outcome.

## 2 Materials and methods

### 2.1 Study design, setting, and reporting

We conducted a multicenter observational cohort study of older adults with aneurysmal subarachnoid hemorrhage (aSAH) in North China. The model-development population comprised admissions to four hospitals between January 2017 and September 2023. A temporally overlapping but institutionally independent cohort recruited between January 2017 and January 2021 was reserved for external validation.

The study was designed as a prediction-model development and external-validation investigation. Reporting was structured with reference to TRIPOD+AI, with particular attention to population flow, outcome definition, predictor timing, model evaluation, and applicability (14). No prediction output was used to alter clinical care in this observational dataset.

### 2.2 Participants

Eligible participants were aged ≥60 years, had a hospital diagnosis of aSAH during the stated recruitment periods, and had ascertainable 2-year functional outcome. The development stream screened 1,132 admissions and excluded 210 patients lost to 2-year follow-up and 8 with unknown aneurysm location, leaving 914 participants. These patients were randomly divided 7:3 into training (*n* = 640) and internal-validation (*n* = 274) cohorts. The external stream screened 602 patients and excluded 96 lost to 2-year follow-up and 7 with unknown aneurysm location, leaving 499 participants (Figure 1).

**Figure 1:**
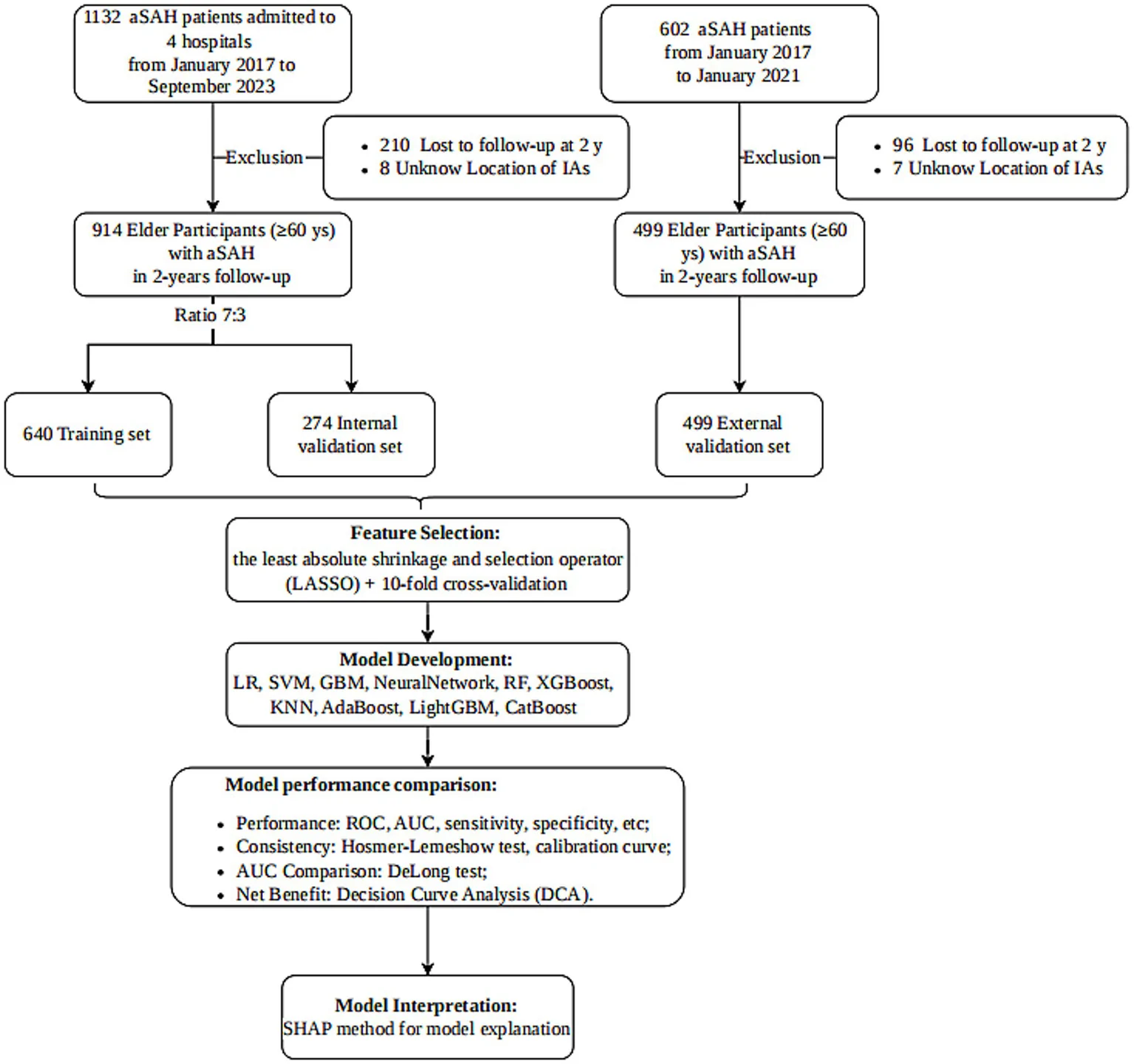
Study population and analytical workflow. The derivation stream screened 1,132 aSAH admissions at four hospitals between January 2017 and September 2023; 914 patients were included in the training and internal-validation cohorts. The independent external stream screened 602 admissions between January 2017 and January 2021 and included 499 patients. LASSO with 10-fold cross-validation preceded comparison of 10 algorithms, calibration, decision-curve analysis, and SHAP interpretation.

All 499 external-validation participants (100%) were enrolled at a single hospital in Shijiazhuang, Hebei Province, a tertiary-grade-A, university-affiliated regional referral center with a 362-bed neurosurgery department (Supplementary Table S2). Because this hospital functions as a regional receiving center, 89.4% of external participants were interhospital referrals, a substantially higher proportion than in the development cohorts, reflecting a distinct care pathway.

### 2.3 Outcome definition and follow-up

The primary outcome was functional status 2 years after aSAH, assessed with the modified Rankin Scale (mRS) (15). Good outcome was defined as mRS 0–2 and poor outcome as mRS 3–6; poor outcome was treated as the positive class for prediction.

Overall survival was examined as an exploratory secondary outcome in the pooled analytic population using the available longitudinal follow-up.

### 2.4 Candidate predictors

Candidate predictors were drawn from demographics, residence and referral information, comorbidity, aneurysm characteristics, admission neurological severity, treatment modality, and health-system context. Neurological severity was represented by Hunt–Hess and World Federation of Neurosurgical Societies (WFNS) grades (16, 17). Aneurysm variables included location, number, and maximum diameter. Treatment was categorized as microsurgical, endovascular, conservative, or combined treatment. Hospital capacity was categorized as low, medium, or high. This classification was based on the structural scale of each participating hospital’s neurosurgery department—the number of dedicated neurosurgical wards and the total number of open neurosurgery beds (Supplementary Table S2). Hospitals with a single general ward and a comparatively small bed complement were classified as low-capacity; hospitals with one dedicated ward plus a neurocritical-care/NICU unit, totaling fewer than 100 beds, were classified as medium-capacity; and hospitals with multiple (≥7) subspecialty wards and more than 300 total beds, including dedicated neurocritical-care capacity, were classified as high-capacity.

The 14 features used in the final GBM interpretation were Hunt–Hess category, age, WFNS category, treatment, aneurysm size category, Admission Tension–Pressure Index (ATPI), hospital capacity, history of stroke, hypertension (HP), aneurysm location, sex, history of tumor, smoking history, and alcohol history. Post-admission or follow-up variables—including in-hospital death, discharge mRS, length of stay, and observed survival/follow-up duration—were excluded from prediction to prevent outcome leakage (Table 1).

**Table 1:** Clinical characteristics, and follow-up characteristics that variables excluded from model between training set, internal validation set and external validation set with good prognosis (2 years mRS 0–2) and poor prognosis (2 years mRS 3–6) that variables excluded from model.

Variable	Training set			Internal validation set			External validation set		
	Good prognosis (*n*=374)	Poor prognosis (*n*=266)	*P* value	Good prognosis (*n*=149)	Poor prognosis (*n*=125)	*P* value	Good prognosis (*n*=307)	Poor prognosis (*n*=192)	*P* value
Perioperative complications									
In-hospital death	0 (0.00%)	69 (25.94%)	<0.001	0 (0.00%)	32 (25.60%)	<0.001	0 (0.00%)	8 (4.20%)	<0.001
Discharge mRS									
mRS 0–2	326 (87.17%)	68 (25.56%)	<0.001	127 (85.23%)	22 (17.60%)	<0.001	262 (85.30%)	43 (22.40%)	<0.001
mRS 3–6	48 (12.83%)	198 (74.44%)		22 (14.77%)	103 (82.40%)		45 (14.70%)	149 (77.60%)	
Length of stay (days) mean±SD	16.64 ± 9.04	14.33 ± 18.43	0.060	16.54 ± 9.00	15.82 ± 18.23	0.688	12.42 ± 7.28	10.44 ± 10.35	<0.001
Follow-up characteristics									
Overall survival time (months) mean±SD	42.78 ± 14.40	11.39 ± 17.54	<0.001	42.83 ± 13.95	13.04 ± 17.43	<0.001	42.30 ± 14.03	15.33 ± 19.65	<0.001
Follow-up time (months) mean±SD	42.56 ± 15.02	27.87 ± 22.73	<0.001	42.66 ± 14.79	31.90 ± 20.74	<0.001	42.23 ± 14.49	28.05 ± 22.56	<0.001

Categorical variables are presented as frequency (percentage) and compared using Pearson chi-squared test or Fisher's exact test. Continuous variables are presented as mean ± standard deviation (SD). A two-tailed *P*-value <0.05 was considered statistically significant. mRS, modified Rankin Scale; SD, standard deviation.

### 2.5 Data preparation and missing data

Data cleaning, variable coding, and preprocessing were completed before model fitting. The development dataset was divided in a 7:3 ratio into training and internal-validation cohorts. Feature selection and model development were conducted within the development data, and the external cohort remained separate until final evaluation.

### 2.6 Feature selection and model development

Least absolute shrinkage and selection operator (LASSO) logistic regression was applied with 10-fold cross-validation (18). Cross-validation identified 14 nonzero features at λ.min and 9 at λ.1se (Supplementary Figure 1). The 14-predictor solution was carried forward for model development and SHAP interpretation.

LASSO was chosen for feature reduction because of its established regularization properties, the interpretability of its coefficient paths, and its suitability for the development-stage sample size, rather than to impose linear structure on the downstream classifier. We acknowledge that, as a linear method, LASSO may down-weight predictors whose association with outcome is primarily nonlinear or expressed through interactions, and could therefore exclude variables that a tree-based selection procedure (e.g., Boruta or SHAP-based recursive feature elimination) would retain for a nonlinear model such as GBM. This two-stage linear-selection/nonlinear-modeling workflow is a methodological limitation of the current analysis.

To empirically test this concern, a sensitivity comparison against a nonlinear, all-relevant feature-selection procedure (Boruta) was performed. Boruta wraps a random-forest importance test: at each of 100 iterations, the Gini importance of every candidate predictor is compared with that of “shadow” features created by randomly permuting each real predictor, and variables are iteratively confirmed, rejected, or left tentative according to whether their importance exceeds the maximum shadow importance. Because the underlying importance measure comes from a random forest, Boruta is sensitive to nonlinear and interaction effects that LASSO’s linear coefficients cannot capture, providing a direct check on whether linear screening had discarded predictors of value to a nonlinear classifier such as GBM. Boruta was applied to the development cohort (*n* = 914) using the same candidate predictor set considered for LASSO (Supplementary Figure 5).

Ten candidate algorithms were evaluated: logistic regression, support vector machine, GBM, neural network, random forest, XGBoost, k-nearest neighbors, AdaBoost, LightGBM, and CatBoost. GBM constructs an additive ensemble of weak learners by sequentially minimizing a loss function (19). All models were developed using a common analytical workflow, and GBM was retained as the principal model for full validation and SHAP interpretation.

### 2.7 Model evaluation

Discrimination was summarized using the area under the receiver operating characteristic curve (AUC) with 95% confidence intervals, accuracy, sensitivity, specificity, precision, and F1 score. Poor 2-year outcome was treated as the positive class. Pairwise AUC differences were explored using DeLong’s method (20); because several confidence intervals overlapped, model comparisons were interpreted using both discrimination and classification metrics.

Calibration was examined graphically by cohort. Decision-curve analysis quantified standardized net benefit across threshold probabilities relative to treat-all and treat-none strategies (21). Because the calibration plots used three risk groups, graphical agreement was interpreted cautiously.

### 2.8 Model interpretation

The final GBM was interpreted with SHAP (22). Global importance was summarized by mean absolute SHAP value; a beeswarm plot displayed the direction and distribution of contributions. Force and waterfall plots illustrated one prediction, and a dependence plot examined the Hunt–Hess/WFNS relationship. SHAP values explain the fitted model and do not establish causal effects.

To move beyond marginal SHAP importance, pairwise SHAP interaction values were computed for the final GBM using TreeSHAP’s interaction-value decomposition, and the ten largest mean absolute pairwise interactions were ranked. The age × Hunt–Hess interaction was examined in detail using an interaction dependence plot (age on the x-axis, SHAP interaction value on the y-axis, colored by Hunt–Hess category) and by stratifying patients into four age (<70/≥70 years) × Hunt–Hess (low I–III/high IV–V) groups, within which mean SHAP interaction values and observed poor-outcome proportions were summarized (Supplementary Figure 4; Supplementary Table S1).

### 2.9 Descriptive and survival analyses

Categorical variables were summarized as count (percentage) and compared using Pearson’s chi-squared test or Fisher’s exact test, as appropriate. Continuous variables were summarized as mean±SD and compared using distribution-appropriate two-sample tests. Kaplan–Meier curves and log-rank tests compared unadjusted survival across treatment and hospital-capacity groups. Two-sided *p* < 0.05 was considered statistically significant. Baseline *P* values were descriptive and were not interpreted as independent effects; no multiplicity adjustment was applied.

To evaluate whether the unadjusted survival differences after accounting for baseline severity and case mix, a multivariable Cox proportional-hazards model for overall survival was fitted in the pooled cohort, with treatment modality (reference: endovascular), hospital capacity (reference: high), Hunt–Hess grade, WFNS grade, age, sex, history of stroke, hypertension, aneurysm location, and aneurysm size as covariates. Robust standard errors clustered by hospital were used to account for center-level correlation, and results are reported as adjusted hazard ratios (aHR) with 95% confidence intervals (Figure 2).

**Figure 2:**
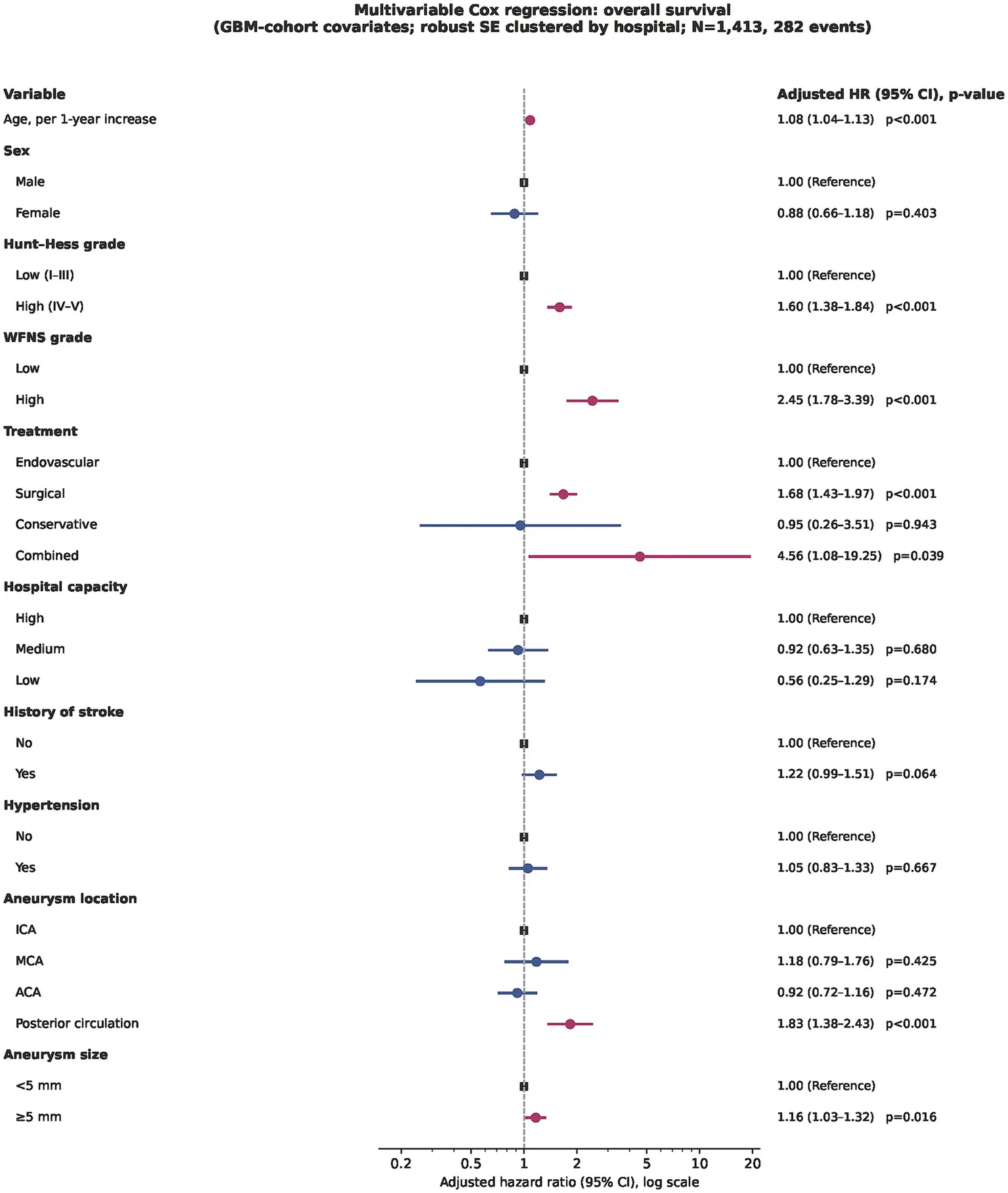
Multivariable Cox proportional-hazards model for overall survival in the pooled cohort.

### 2.10 Ethics

The study was conducted in accordance with the Declaration of Helsinki and applicable institutional requirements. The study investigators are responsible for providing the approving ethics committee name, approval identifier, and informed-consent or waiver statement during journal submission.

## 3 Results

### 3.1 Study population and 2-year outcomes

The study included 1,413 participants: 640 in the training cohort, 274 in the internal-validation cohort, and 499 in the external-validation cohort (Figure 1). Poor 2-year functional outcome occurred in 266/640 (41.6%), 125/274 (45.6%), and 192/499 (38.5%), respectively, for 583/1,413 (41.3%) overall. Good outcome occurred in 830 (58.7%). The displayed flow counts indicate that 306 of 1,734 screened patients were lost to 2-year follow-up and 15 had unknown aneurysm location.

Patients with poor outcomes were older in all three cohorts: 71.07 ± 7.55 versus 67.09 ± 4.84 years in training, 71.38 ± 7.62 versus 66.87 ± 5.13 years in internal validation, and 70.44 ± 6.34 versus 66.79 ± 4.98 years in external validation (all *p* < 0.001; Table 2). High Hunt–Hess grades (IV–V) occurred in 50.8, 48.8, and 42.2% of poor-outcome patients compared with 8.6, 10.1, and 7.8% of good-outcome patients (all *p* < 0.001). Corresponding high WFNS grades occurred in 60.5, 66.4, and 56.3% versus 16.3, 18.1, and 17.9% (all *p* < 0.001).

**Table 2:** Demographic and clinical characteristics between training set, internal validation set and external validation set with good prognosis (2 years mRS 0–2) and poor prognosis (2 years mRS 3–6).

Variable	Training set			Internal validation set			External validation set		
	Good prognosis (*n*=374)	Poor prognosis (*n*=266)	*P* value	Good prognosis (*n*=149)	Poor prognosis (*n*=125)	*P* value	Good prognosis (*n*=307)	Poor prognosis (*n*=192)	*P* value
Demographic characteristics									
Sex									
Male	89 (23.80%)	71 (26.69%)	0.405	46 (30.87%)	31 (24.80%)	0.265	81 (26.38%)	60 (31.25%)	0.240
Female	285 (76.20%)	195 (73.31%)		103 (69.13%)	94 (75.20%)		226 (73.62%)	132 (68.75%)	
Age									
Mean ± SD	67.09 ± 4.84	71.07 ± 7.55	<0.001	66.87 ± 5.13	71.38 ± 7.62	<0.001	66.79 ± 4.98	70.44 ± 6.34	<0.001
Patient's residence									
Non-urban	293 (78.34%)	215 (80.83%)	0.444	115 (77.18%)	103 (82.40%)	0.286	222 (72.31%)	144 (75.00%)	0.509
Urban	81 (21.66%)	51 (19.17%)		34 (22.82%)	22 (17.60%)		85 (27.69%)	48 (25.00%)	
ATPI									
Low	131 (35.03%)	81 (30.45%)	0.180	51 (34.23%)	50 (40.00%)	0.602	97 (31.60%)	57 (29.69%)	0.440
Middle	136 (36.36%)	91 (34.21%)		59 (39.60%)	44 (35.20%)		117 (38.11%)	84 (43.75%)	
High	107 (28.61%)	94 (35.34%)		39 (26.17%)	31 (24.80%)		93 (30.29%)	51 (26.56%)	
Capacity of hospital									
Low	48 (12.83%)	69 (25.94%)	<0.001	32 (21.48%)	30 (24.00%)	0.042	0 (0.00%)	0 (0.00%)	-
Middle	215 (57.49%)	129 (48.50%)		81 (54.36%)	50 (40.00%)		0 (0.00%)	0 (0.00%)	
High	111 (29.68%)	68 (25.56%)		36 (24.16%)	45 (36.00%)		307 (100.00%)	192 (100.00%)	
Clinical characteristics									
Medical history									
Hypertension	254 (67.91%)	200 (75.19%)	0.046	99 (66.44%)	97 (77.60%)	0.042	206 (67.10%)	144 (75.00%)	0.061
Diabetes mellitus	25 (6.68%)	25 (9.40%)	0.207	13 (8.72%)	8 (6.40%)	0.471	26 (8.47%)	16 (8.33%)	0.958
Dyslipidaemia	11 (2.94%)	13 (4.89%)	0.202	3 (2.01%)	7 (5.60%)	0.115	2 (0.65%)	2 (1.04%)	0.634
Stroke	39 (10.43%)	52 (19.55%)	0.001	13 (8.72%)	24 (19.20%)	0.012	31 (10.10%)	32 (16.67%)	0.032
Tumor	4 (1.07%)	11 (4.14%)	0.012	6 (4.03%)	2 (1.60%)	0.235	9 (2.93%)	5 (2.60%)	0.829
CVD	38 (10.16%)	39 (14.66%)	0.085	17 (11.41%)	27 (21.60%)	0.022	38 (12.38%)	35 (18.23%)	0.072
Habits									
Tobacco history	31 (8.29%)	33 (12.41%)	0.087	18 (12.08%)	6 (4.80%)	0.034	16 (5.21%)	17 (8.85%)	0.111
Alcohol history	16 (4.28%)	17 (6.39%)	0.234	14 (9.40%)	8 (6.40%)	0.363	10 (3.26%)	8 (4.17%)	0.596
Location of IAs									
ICA	216 (57.75%)	131 (49.25%)	0.055	76 (51.01%)	62 (49.60%)	0.003	173 (56.35%)	105 (54.69%)	0.018
MCA	50 (13.37%)	32 (12.03%)		22 (14.77%)	19 (15.20%)		34 (11.07%)	25 (13.02%)	
ACA	70 (18.72%)	61 (22.93%)		42 (28.19%)	20 (16.00%)		74 (24.10%)	31 (16.15%)	
PCircA	38 (10.16%)	42 (15.79%)		9 (6.04%)	24 (19.20%)		26 (8.47%)	31 (16.15%)	
Number of IAs									
1	283 (75.67%)	198 (74.44%)	0.722	118 (79.19%)	90 (72.00%)	0.165	250 (81.43%)	146 (76.04%)	0.148
≥2	91 (24.33%)	68 (25.56%)		31 (20.81%)	35 (28.00%)		57 (18.57%)	46 (23.96%)	
Maximum aneurysm diameter									
<5mm	198 (52.94%)	110 (41.35%)	0.004	80 (53.69%)	57 (45.60%)	0.182	154 (50.16%)	86 (44.79%)	0.243
≥5mm	176 (47.06%)	156 (58.65%)		69 (46.31%)	68 (54.40%)		153 (49.84%)	106 (55.21%)	
Hunt-Hess scale									
I-III	342 (91.44%)	131 (49.25%)	<0.001	134 (89.93%)	64 (51.20%)	<0.001	283 (92.18%)	111 (57.81%)	<0.001
IV-V	32 (8.56%)	135 (50.75%)		15 (10.07%)	61 (48.80%)		24 (7.82%)	81 (42.19%)	
WFNS grade									
I-III	313 (83.69%)	105 (39.47%)	<0.001	122 (81.88%)	42 (33.60%)	<0.001	252 (82.08%)	84 (43.75%)	<0.001
IV-V	61 (16.31%)	161 (60.53%)		27 (18.12%)	83 (66.40%)		55 (17.92%)	108 (56.25%)	
Treatment									
Surgical treatment	96 (25.67%)	60 (22.56%)	<0.001	43 (28.86%)	38 (30.40%)	<0.001	112 (36.48%)	50 (26.04%)	<0.001
Endovascular treatment	255 (68.18%)	99 (37.22%)		91 (61.07%)	50 (40.00%)		176 (57.33%)	91 (47.40%)	
Conservative treatment	23 (6.15%)	104 (39.10%)		15 (10.07%)	37 (29.60%)		18 (5.86%)	51 (26.56%)	
Combine	0 (0.00%)	3 (1.13%)		0 (0.00%)	0 (0.00%)		1 (0.33%)	0 (0.00%)	
Time for symptom onset to treatment (hours) mean±SD	32.44 ± 83.23	33.72 ± 269.14	0.931	27.12 ± 62.21	16.05 ± 44.36	0.088	53.29 ± 102.92	33.64 ± 51.04	0.005
Referral	192 (51.34%)	133 (50.00%)	0.739	70 (46.98%)	79 (63.20%)	0.007	277 (90.23%)	169 (88.02%)	0.436

Categorical variables are presented as frequency (percentage) and compared using Pearson chi-squared test or Fisher's exact test. Continuous variables are presented as mean ± standard deviation (SD). A two-tailed *P*-value <0.05 was considered statistically significant. mRS, modified Rankin Scale; IAs, intracranial aneurysms; ICA, internal carotid artery; MCA, middle cerebral artery; ACA, anterior cerebral artery; PCircA, posterior circulation artery; WFNS, World Federation of Neurosurgical Societies; CVD, cardiovascular disease; IQR, interquartile range; SD, standard deviation; ATPI, Admission Tension-Pressure Index.

Treatment distributions differed by 2-year outcome in all cohorts (all *p* < 0.001). Prior stroke was more frequent in the poor-outcome groups (training 19.6% vs. 10.4%, *p* = 0.001; internal validation 19.2% vs. 8.7%, *p* = 0.012; external validation 16.7% vs. 10.1%, *p* = 0.032). Aneurysm location differed by outcome in internal and external validation (*p* = 0.003 and *p* = 0.018) but not in training (*p* = 0.055). Maximum aneurysm diameter differed only in training (*p* = 0.004). Hospital-capacity distribution differed in training (*p* < 0.001) and internal validation (*p* = 0.042), but it could not be contrasted in external validation because every patient was treated in a high-capacity hospital. Referral was substantially more common externally (89.4% overall) than in the development cohorts, indicating a distinct care pathway.

Post-baseline characteristics were associated with 2-year status but were excluded from prediction (Table 1). In-hospital deaths occurred only in the poor-outcome group, and discharge mRS was strongly associated with 2-year outcome (all *p* < 0.001). Length of stay did not differ significantly in training or internal validation (*p* = 0.060 and *p* = 0.688), whereas a difference was reported externally (12.42 ± 7.28 vs. 10.44 ± 10.35 days, *p* < 0.001). Overall survival and observed follow-up durations were longer among participants with good 2-year outcomes (all *p* < 0.001).

### 3.2 Feature selection

Ten-fold cross-validation of LASSO showed 14 retained predictors at λ.min and 9 at λ.1se (Supplementary Figure 1). The 14-predictor solution used in the subsequent GBM analyses comprised age, sex, Hunt–Hess and WFNS categories, treatment, aneurysm size and location, ATPI, hospital capacity, prior stroke, hypertension, tumor history, smoking history, and alcohol history.

The Boruta sensitivity analysis confirmed seven predictors as robustly important across 100 iterations: WFNS grade, Hunt–Hess grade, treatment modality, and age (importance well above the maximum shadow feature), followed by hospital capacity, aneurysm location, and history of stroke (confirmed just above the shadow threshold); no predictor was left tentative. Every Boruta-confirmed predictor was also selected by LASSO, and LASSO additionally retained seven further predictors (aneurysm size, cardiovascular disease, ATPI, hypertension, sex, smoking history, and alcohol history) that Boruta did not confirm as important (Supplementary Figure 5). Thus, LASSO screening was, if anything, more permissive than the nonlinear Boruta procedure and did not exclude any predictor independently identified as important by a method sensitive to nonlinear and interaction effects.

### 3.3 Comparative model performance

Apparent training AUCs ranged from 0.794 for AdaBoost to 0.948 for random forest (Table 3; Figure 3). Random forest, k-nearest neighbors, and LightGBM had very high training AUCs (0.948, 0.940, and 0.936) but lower internal-validation AUCs (0.839, 0.794, and 0.773) and external-validation AUCs (0.787, 0.752, and 0.693), consistent with limited transportability or overfitting.

**Table 3:** Comparative analysis of the performance outcomes across 10 machine learning models.

Model	Accuracy	Sensitivity	Specificity	Precision	F1 score	AUC
Training set						
Logistic	0.766	0.818	0.728	0.691	0.749	0.840
SVM	0.768	0.850	0.706	0.683	0.758	0.844
GBM	0.796	0.777	0.809	0.753	0.765	0.863
NeuralNetwork	0.771	0.832	0.725	0.693	0.756	0.842
RandomForest	0.878	0.847	0.902	0.866	0.856	0.948
Xgboost	0.782	0.796	0.771	0.722	0.757	0.862
KNN	0.850	0.923	0.796	0.771	0.841	0.940
Adaboost	0.746	0.763	0.733	0.681	0.719	0.794
LightGBM	0.886	0.807	0.946	0.917	0.858	0.936
CatBoost	0.778	0.796	0.766	0.717	0.754	0.848
Internal validation set						
Logistic	0.766	0.769	0.763	0.709	0.738	0.813
SVM	0.758	0.778	0.744	0.695	0.734	0.820
GBM	0.780	0.709	0.833	0.761	0.735	0.834
NeuralNetwork	0.769	0.744	0.788	0.725	0.734	0.794
RandomForest	0.762	0.838	0.705	0.681	0.751	0.839
Xgboost	0.773	0.778	0.769	0.717	0.746	0.832
KNN	0.758	0.769	0.750	0.698	0.732	0.794
Adaboost	0.703	0.769	0.654	0.625	0.690	0.749
LightGBM	0.736	0.744	0.731	0.674	0.707	0.773
CatBoost	0.791	0.692	0.865	0.794	0.740	0.838
External validation set						
Logistic	0.735	0.693	0.762	0.646	0.668	0.785
SVM	0.725	0.734	0.720	0.621	0.673	0.785
GBM	0.752	0.661	0.808	0.683	0.672	0.791
NeuralNetwork	0.721	0.724	0.720	0.618	0.667	0.784
RandomForest	0.737	0.698	0.762	0.647	0.672	0.787
Xgboost	0.735	0.714	0.749	0.640	0.675	0.770
KNN	0.745	0.505	0.896	0.752	0.604	0.752
Adaboost	0.703	0.656	0.733	0.606	0.630	0.723
LightGBM	0.717	0.542	0.827	0.662	0.596	0.693
CatBoost	0.731	0.698	0.752	0.638	0.667	0.780

SVM, support vector machine; GBM, gradient boosting machine; KNN, k-nearest neighbors; LightGBM, light gradient boosting machine; AUC, area under the receiver operating characteristic curve.

**Figure 3:**
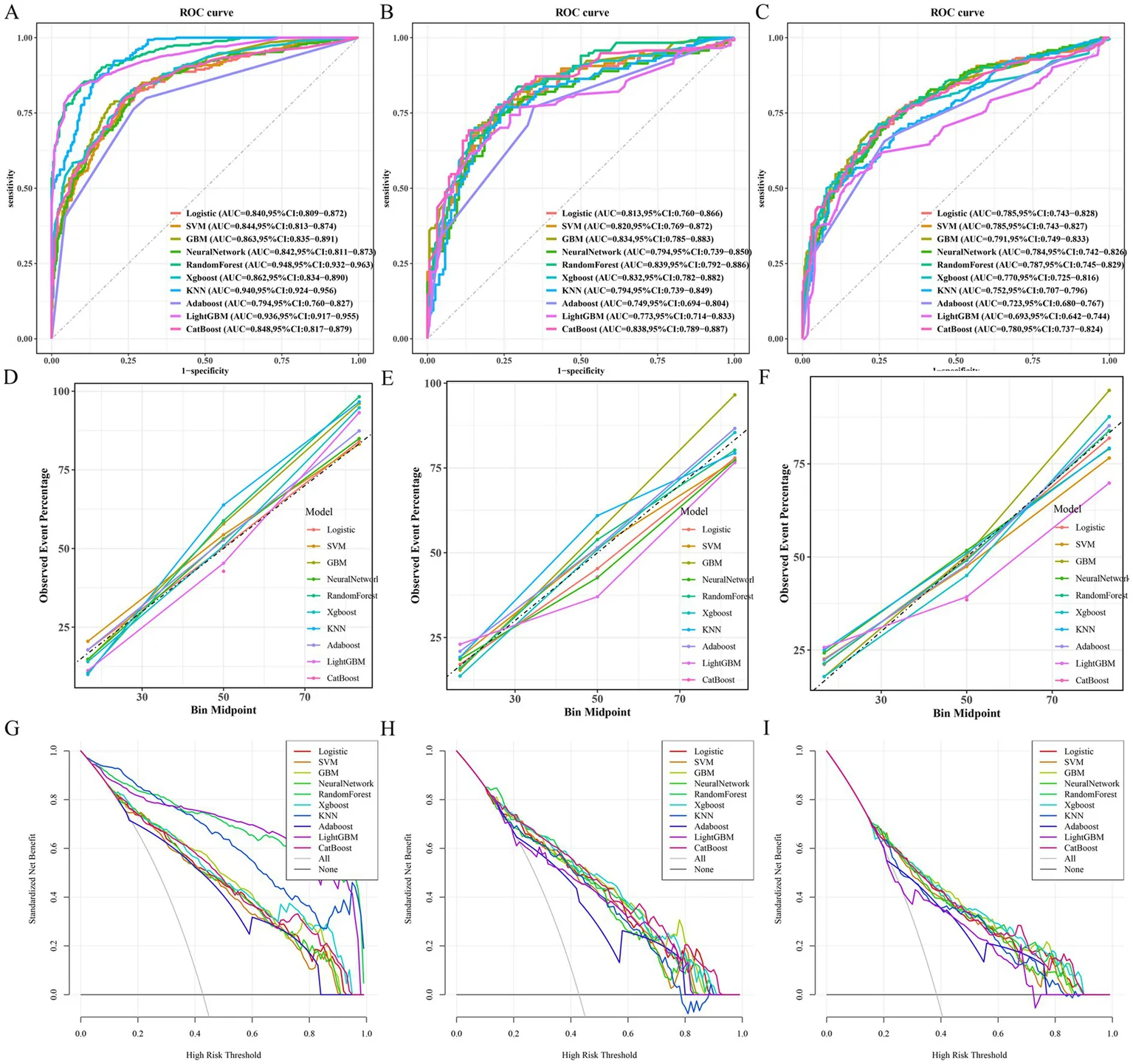
Performance of 10 machine-learning models in the training, internal-validation, and external-validation cohorts. **(A–C)** Receiver operating characteristic curves. **(D–F)** Calibration plots. **(G–I)** Decision-curve analyses, with treat-all and treat-none reference strategies.

GBM discrimination was comparatively stable: AUC 0.863 (95% CI, 0.835–0.891) in training, 0.834 (0.785–0.883) in internal validation, and 0.791 (0.749–0.833) externally. In internal validation, random forest had the highest AUC (0.839) and F1 score (0.751), whereas CatBoost had the highest accuracy (0.791), specificity (0.865), and precision (0.794). GBM accuracy was 0.780, sensitivity 0.709, specificity 0.833, precision 0.761, and F1 score 0.735.

In external validation, GBM had the highest AUC and accuracy point estimates among the 10 candidates (0.791 and 0.752), with sensitivity 0.661, specificity 0.808, precision 0.683, and F1 score 0.672 (Table 4). It did not lead all classification metrics: support vector machine had the highest sensitivity (0.734), k-nearest neighbors the highest specificity and precision (0.896 and 0.752), and XGBoost the highest F1 score (0.675). The GBM AUC confidence interval overlapped those of several alternatives; therefore, statistical superiority is not claimed.

**Table 4:** Performance of GBM models with different feature combinations on the internal validation set and external validation set.

Model	Internal validation set	External validation set
Accuracy	0.780	0.752
Sensitivity	0.709	0.661
Specificity	0.833	0.808
Precision	0.761	0.683
F1 score	0.735	0.672
AUC	0.834	0.791

GBM, gradient boosting machine; AUC, area under the receiver operating characteristic curve.

### 3.4 Calibration and decision-curve analysis

Calibration curves showed broadly similar observed and predicted event rates around the middle risk range, with larger deviations in the tails (Figures 3D–F). Decision curves suggested positive standardized net benefit for several models across portions of the clinically relevant threshold range (Figures 3G–I). External curves were closely clustered and became unstable at high thresholds; GBM was often among the upper curves in the middle range but did not show unequivocal dominance.

### 3.5 Explainability of the GBM

Global SHAP importance ranked Hunt–Hess category, age, and WFNS category as the three dominant contributors, followed by treatment (Figures 4A,B). Aneurysm size, ATPI, hospital capacity, prior stroke, HP, aneurysm location, sex, tumor history, smoking, and alcohol contributed substantially less on average. Higher-coded Hunt–Hess and WFNS values and older age generally shifted predictions toward poor outcome, whereas the direction of multicategory features depended on their encoding.

**Figure 4:**
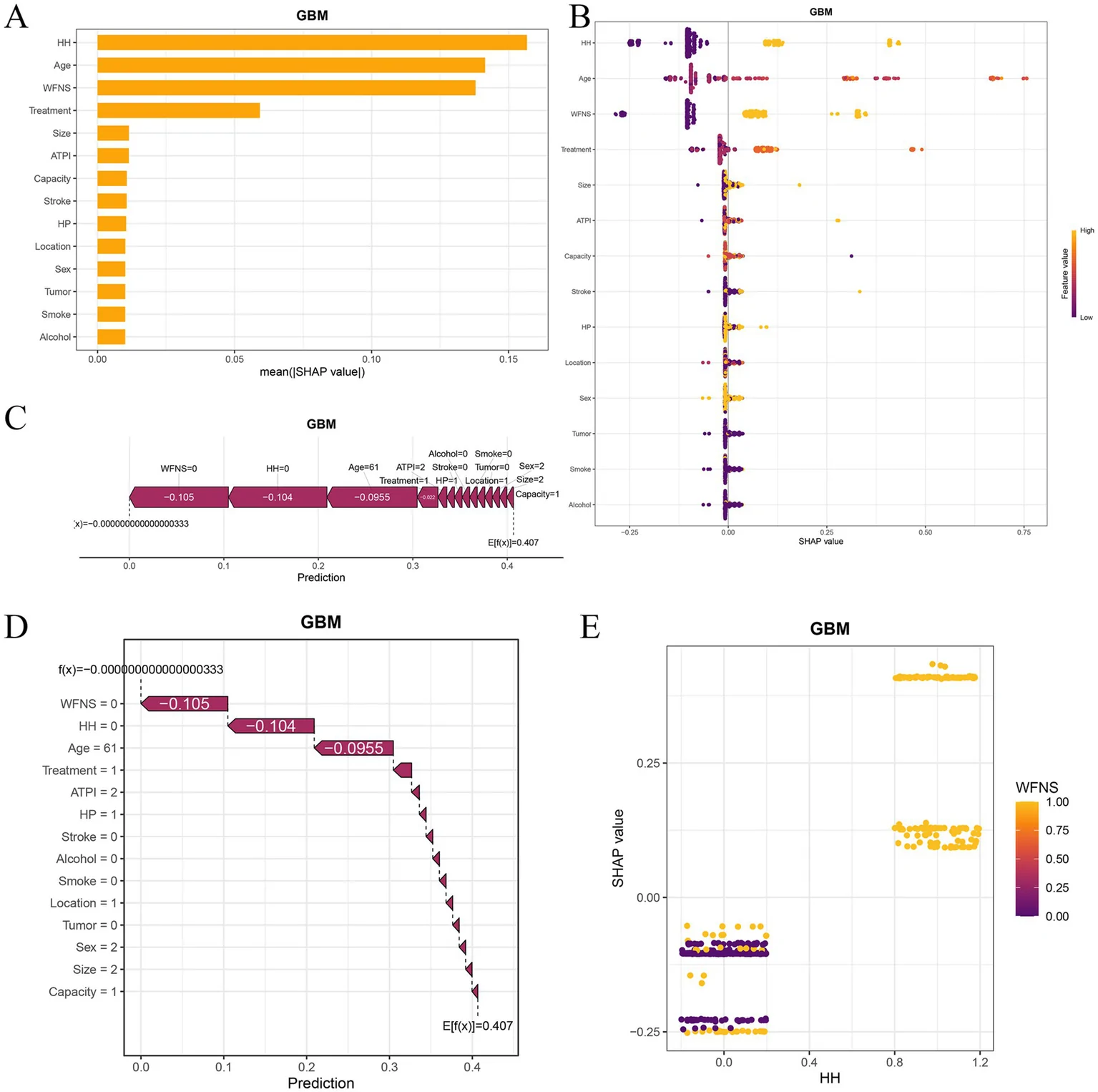
SHAP-based interpretation of the GBM. **(A)** Mean absolute SHAP values ranked global feature importance. **(B)** Beeswarm plot showing the direction and distribution of feature contributions. **(C,D)** Force and waterfall plots for an individual prediction. **(E)** Dependence plot for Hunt–Hess category colored by WFNS category.

For the illustrated individual aged 61 years with low-coded Hunt–Hess and WFNS values, the model output was reduced from a baseline of 0.407 to approximately zero; the largest negative contributions came from WFNS, Hunt–Hess, and age (Figures 4C,D). The Hunt–Hess dependence plot showed negative contributions for the low-coded category and positive contributions for the high-coded category (Figure 4E). Because high Hunt–Hess and high WFNS values were strongly co-occurring, this plot does not independently establish a biological interaction.

SHAP interaction values confirmed Hunt–Hess × age as the strongest pairwise interaction in the GBM (mean |SHAP interaction value| ≈ 0.11), ahead of age × treatment, Hunt–Hess × Admission Tension–Pressure Index, and age × WFNS (Supplementary Figure 4A). The age × Hunt–Hess interaction dependence plot showed a systematic divergence by severity category with advancing age: among patients with low Hunt–Hess grade the interaction value remained close to zero across the age range, whereas among patients with high Hunt–Hess grade the interaction value was strongly positive at younger ages and became negative beyond approximately 75–80 years (Supplementary Figure 4B). Stratifying the cohort into four age–severity groups showed that the two age–severity–concordant strata carried a positive mean interaction value together with the highest observed poor-outcome rates: younger patients with low Hunt–Hess grade (*n* = 701; poor outcome 20.3%; mean interaction +0.068) and, most markedly, older patients with high Hunt–Hess grade (*n* = 159; poor outcome 90.6%; mean interaction +0.170). The two age–severity–discordant strata carried negative mean interaction values—younger patients with high Hunt–Hess grade (*n* = 189; poor outcome 70.4%; mean interaction −0.208) and older patients with low Hunt–Hess grade (*n* = 364; poor outcome 45.1%; mean interaction −0.074)—indicating that the model partly offset the risk attributable to age or severity alone when the two were discordant, plausibly reflecting greater physiological reserve in younger high-grade patients (Supplementary Figure 4C; Supplementary Table S1). This pattern extends the marginal SHAP ranking of Hunt–Hess, age, and WFNS by showing that the model’s weighting of neurological severity is age-dependent rather than fixed; as a model-derived quantity, the SHAP interaction value is not equivalent to a statistically tested interaction term and should be regarded as hypothesis-generating.

### 3.6 Exploratory survival patterns

At time zero, the pooled survival analysis included 399 surgically treated, 762 endovascularly treated, 248 conservatively treated, and 4 combined-treatment patients; hospital-capacity groups included 179 low-, 475 medium-, and 759 high-capacity patients (Figure 5). Unadjusted survival differed across treatment modalities and capacity categories (both global log-rank *p* < 0.0001). Conservative treatment and low hospital capacity were associated with lower observed survival. These curves were not adjusted for neurological severity, frailty, referral selection, treatment indication, center clustering, or competing care pathways. The combined-treatment stratum and late follow-up tails were too sparse for reliable interpretation.

**Figure 5:**
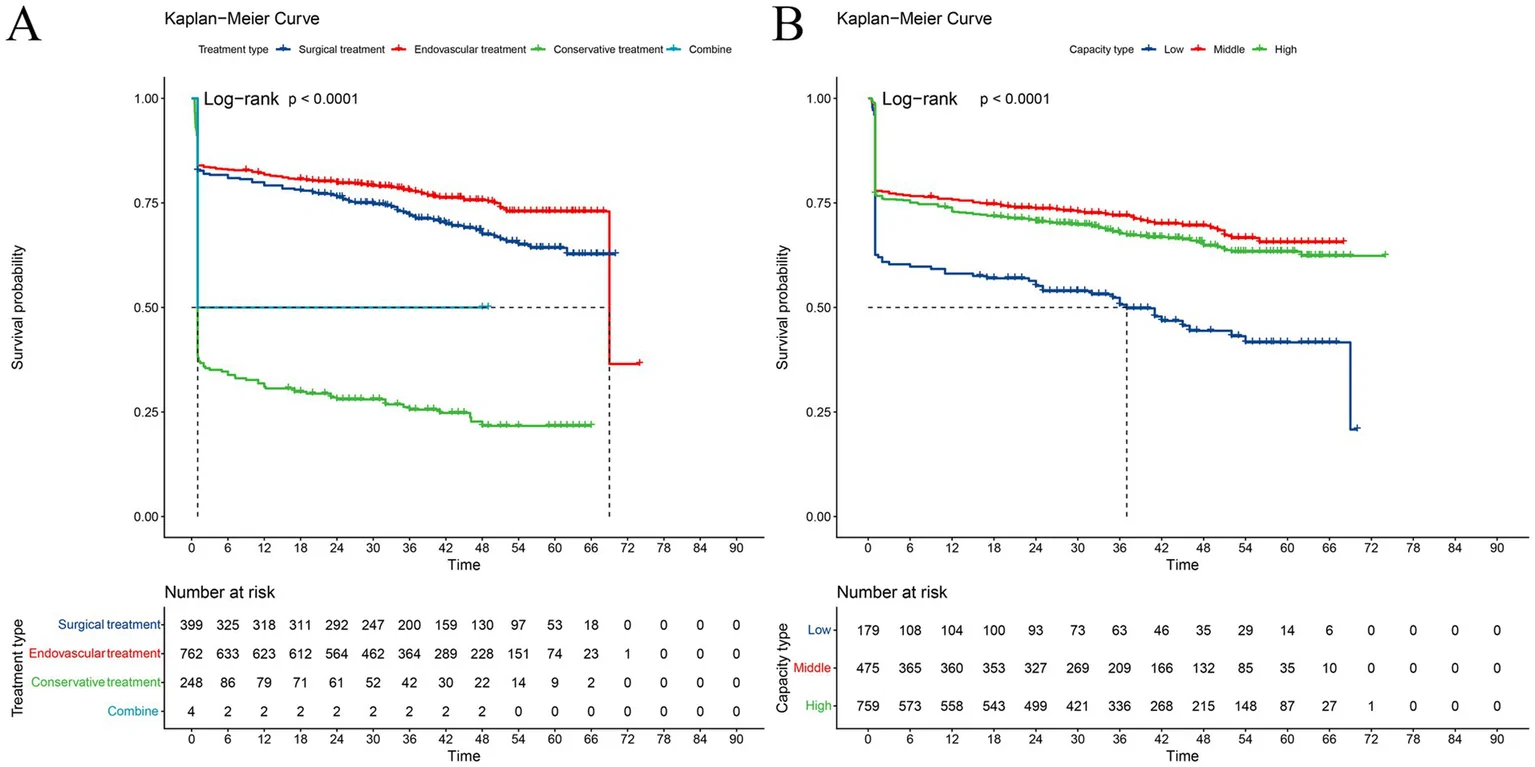
Kaplan–Meier survival curves by treatment and hospital capacity. **(A)** Surgical, endovascular, conservative, and combined treatment groups. **(B)** Low-, medium-, and high-capacity hospitals. Numbers at risk are shown below each panel. Curves are unadjusted and should not be interpreted as causal treatment or capacity effects.

A multivariable Cox proportional-hazards model for overall survival was fitted in the pooled cohort (*N* = 1,413; 282 events), adjusting for age, sex, Hunt–Hess grade, WFNS grade, treatment modality, hospital capacity, history of stroke, hypertension, aneurysm location, and aneurysm size, with robust standard errors clustered by hospital (Figure 2). After adjustment, age (adjusted hazard ratio [aHR] 1.08 per year, 95% CI 1.04–1.13, *p* < 0.001), high Hunt–Hess grade (aHR 1.60, 1.38–1.84, *p* < 0.001), high WFNS grade (aHR 2.45, 1.78–3.39, *p* < 0.001), posterior-circulation aneurysm location (aHR 1.83, 1.38–2.43, *p* < 0.001), and aneurysm size ≥5 mm (aHR 1.16, 1.03–1.32, *p* = 0.016) remained independently associated with mortality. Compared with endovascular treatment, surgical treatment remained independently associated with higher hazard after adjustment (aHR 1.68, 95% CI 1.43–1.97, *p* < 0.001); the combined-treatment estimate was imprecise given only four patients in that group (aHR 4.56, 1.08–19.25, *p* = 0.039), and conservative treatment was not significantly different from endovascular treatment (aHR 0.95, 0.26–3.51, *p* = 0.943). In contrast, hospital capacity was no longer significantly associated with survival after adjustment (medium vs. high: aHR 0.92, 0.63–1.35, *p* = 0.680; low vs. high: aHR 0.56, 0.25–1.29, *p* = 0.174), and history of stroke showed only a borderline association (aHR 1.22, 0.99–1.51, *p* = 0.064); sex and hypertension were not significant. These adjusted estimates are reported alongside, rather than instead of, the unadjusted log-rank comparisons above: the hospital-capacity gradient attenuated toward the null after adjustment, whereas the survival disadvantage associated with surgical treatment persisted.

## 4 Discussion

In this multicenter North China cohort of 1,413 adults aged ≥60 years with aSAH, four findings were most important. First, poor functional outcome at 2 years was common, affecting 41.3% overall. Second, an explainable GBM maintained moderate discrimination across internal and external validation, with AUC decreasing from 0.863 in training to 0.834 and 0.791. Third, neurological severity and age dominated model explanations, while treatment and health-system variables contributed more modestly. Fourth, treatment modality and hospital capacity were associated with unadjusted survival, but the data do not identify causal effects. Together, these findings support transparent admission-stage risk stratification while emphasizing that implementation must account for referral structure and hospital context.

The observed importance of Hunt–Hess and WFNS categories is clinically coherent. Both summarize early neurological injury and have long been used for aSAH severity classification (16, 17). Age also showed a consistent relationship with 2-year outcome across all cohorts and remained prominent in SHAP analyses. These results align with established prognostic models, including elderly-specific and SAHIT models, in which age and neurological grade are central (5–7). The machine-learning contribution is therefore not the discovery of an unexpected causal factor, but the combination of familiar signals within a nonlinear prediction function and the demonstration of performance in a separate cohort.

External validation materially changed the model comparison. Random forest, k-nearest neighbors, and LightGBM showed large gaps between training and external AUC, whereas GBM lost less discrimination. Similar findings in prediction research illustrate why high apparent performance should not determine adoption (11). Several external AUCs were close and their confidence intervals overlapped; accordingly, GBM should be described as having the highest external point estimate rather than proven superiority.

The external cohort was not merely a larger sample from the same distribution. Every external participant was treated in a high-capacity hospital, and nearly 90% were referred. In contrast, the development data spanned low-, medium-, and high-capacity settings. This domain shift is informative: the model retained discrimination, but the independent contribution and calibration of hospital capacity could not be tested externally. Before public-health deployment, calibration should be examined within center, capacity, sex, age, residence, and referral strata, and recalibration should be planned when the receiving population differs.

Notably, once Hunt–Hess/WFNS grade, age, treatment, comorbidity, and aneurysm characteristics were accounted for in the adjusted Cox model (Figure 2), the crude survival gradient across hospital-capacity categories was substantially attenuated and no longer statistically significant, suggesting that much of the unadjusted association reflected differences in case severity and referral case-mix rather than an independent effect of capacity per se. This does not exclude a smaller true capacity effect that the present sample was underpowered to detect, particularly for the low-capacity stratum, which contributed comparatively few events.

Because every external-validation patient was treated at a high-capacity hospital and the great majority were referred, external validation in this study demonstrates model performance within this high-capacity, referral-hospital subpopulation rather than across the full range of hospital-capacity settings represented in the development cohort. Generalizability to low- and medium-capacity, non-referral settings therefore remains unverified and should not be assumed.

From a health-system perspective, a transparent risk score could support several actions: early escalation to neurovascular and neurocritical expertise, prioritized transfer when local capability is limited, anticipatory rehabilitation planning, structured family communication, and targeted post-discharge follow-up. These actions are different from using a high predicted risk to limit treatment. Treatment was both a predictor and a marker of selection, severity, anatomy, clinician judgment, and access; unadjusted survival curves therefore cannot estimate the benefit of surgery, endovascular therapy, or hospital capacity. Prior multicenter work in older and poor-grade aSAH similarly underscores the importance of careful case-mix adjustment (4, 23).

The Kaplan–Meier analysis also suggested poorer long-term survival after surgical treatment than after endovascular treatment (Figure 5A), a pattern that should be interpreted cautiously given its unadjusted, exploratory nature. In the randomized Barrow Ruptured Aneurysm Trial, long-term functional outcomes did not differ significantly between clipping and coiling for anterior-circulation aneurysms (24). A plausible explanation for the discrepancy is confounding by indication: in this observational cohort, older patients with more severe neurological grades, larger or more complex aneurysms, or greater comorbidity burden may have been preferentially selected for surgical treatment, whereas randomization in BRAT balanced these factors between arms. Referral patterns, center-level surgical experience, and unmeasured frailty may further contribute.

The completed adjusted analysis showed that the survival disadvantage associated with surgical treatment persisted after accounting for Hunt–Hess/WFNS grade, age, comorbidity, aneurysm characteristics, and hospital clustering (aHR 1.68, 95% CI 1.43–1.97; Figure 2), indicating that the measured severity and case-mix confounders included here do not fully explain the discrepancy with BRAT. Because this remains an observational adjustment rather than a randomized comparison, residual confounding by unmeasured factors—aneurysm complexity and morphology beyond the size and location captured in this model, frailty, functional reserve, and center- or surgeon-level experience and case selection that cannot be excluded, and a causal treatment effect should not be inferred from this analysis alone.

Explainability improved auditability but did not eliminate uncertainty. SHAP ranked the fitted model’s features and made individual contributions visible, yet it cannot distinguish causation from correlation. Categorical features were interpreted at the level of their encoded categories, and individual explanations should therefore be interpreted cautiously. Moreover, a dependence plot with strong co-occurrence between high Hunt–Hess and high WFNS categories cannot by itself establish interaction.

This study has several strengths: a large older-adult cohort, multicenter development, an independent external validation population, comparison of diverse algorithms, and the combined assessment of discrimination, calibration, decision utility, and explainability. The 2-year horizon is clinically meaningful and extends beyond discharge outcomes. The explicit health-system variables also create a foundation for studying model transportability across care environments.

The novelty of this work lies not in identifying previously unrecognized prognostic factors, but in three complementary contributions: (i) external validation of an explainable prediction model specifically in adults aged ≥60 years with aSAH in China, a population with limited prior external-validation evidence; (ii) explicit incorporation of hospital capacity and treatment pathway as health-system-level variables alongside individual clinical predictors, situating risk estimation within the referral structure of North Chinese neurovascular care; and (iii) joint reporting of discrimination, calibration, decision-curve utility, and SHAP-based explainability, which together provide a more complete and auditable basis for admission-stage risk communication than discrimination alone.

The SHAP interaction analysis adds a further, more specific observation to the established relationship between age and neurological grade: the model’s joint, non-additive contribution of age and Hunt–Hess grade was not uniform across combinations of the two. It was most strongly positive—indicating amplification beyond either factor’s marginal contribution—in older patients presenting with high-grade injury, the stratum with the highest observed poor-outcome rate (90.6%). Conversely, younger patients with high-grade injury and older patients with low-grade injury—two age–severity-discordant combinations—showed negative interaction values, consistent with partial offsetting of the risk predicted from age or grade alone. This pattern goes beyond the marginal SHAP ranking of Hunt–Hess, age, and WFNS as top predictors, which by itself largely restates established prognostic knowledge, by suggesting that the clinical weight assigned to Hunt–Hess grade may reasonably differ by patient age rather than following a fixed, age-independent severity threshold. This implication is hypothesis-generating and warrants prospective confirmation, since a SHAP interaction value describes the structure of the fitted model rather than a directly tested statistical interaction term.

The study has several limitations. The observational design is vulnerable to selection bias, confounding by indication, unmeasured frailty and comorbidity, and center-level clustering. A total of 306 screened patients were lost to 2-year follow-up, creating potential attrition bias. The external cohort was restricted to high-capacity hospitals and had an earlier, overlapping calendar period. The binary mRS endpoint loses information across the disability spectrum. Detailed calibration estimates, prospective temporal validation, adjusted survival effects, decision-analytic confidence intervals, fairness assessment, and evidence that use of the model improves care were not available. Finally, the combined-treatment survival group contained only four patients, and late Kaplan–Meier estimates were based on sparse risk sets.

In addition, feature selection used a linear method (LASSO) ahead of a nonlinear classifier (GBM), which may have excluded predictors with primarily nonlinear or interactive associations with outcome.

A *post hoc* Boruta sensitivity analysis was performed to test this directly. Every predictor that Boruta confirmed as robustly important using a nonlinear, random-forest-based importance test—WFNS grade, Hunt–Hess grade, treatment modality, age, hospital capacity, aneurysm location, and history of stroke—had also been retained by LASSO, and LASSO in fact selected seven further predictors beyond this confirmed set (Supplementary Figure 5). This indicates that linear screening did not discard any variable independently identified as important by a method capable of capturing nonlinear or interaction effects, which mitigates, though does not eliminate, concern about the two-stage workflow; a head-to-head comparison of GBM performance using Boruta-selected versus LASSO-selected feature sets was not undertaken and remains a direction for future work.

Future work should use a prospectively specified analytical protocol, report calibration intercepts, slopes, and Brier scores with uncertainty, and examine performance in lower-capacity and non-referral settings. Temporal and geographic validation, subgroup fairness assessment, and prospective impact studies are needed to determine whether model-guided escalation or follow-up improves patient-centered outcomes without increasing inequity. In particular, because the present external cohort was restricted to high-capacity, predominantly referral hospitals, future validation should prioritize low- and medium-capacity, non-referral settings to establish whether model performance is transportable across the full spectrum of care environments represented in the development cohort.

## 5 Conclusion

An explainable GBM predicted poor 2-year functional outcome among older adults with aSAH with moderate discrimination in an external North China cohort. Admission neurological severity and age were the principal model contributors, while treatment and hospital capacity placed predictions within a broader care-system context. The model may support triage, referral, communication, and longitudinal care planning, but it should not be used to infer treatment effects or withhold care. Prospective recalibration, fairness evaluation, and clinical-impact testing are required before implementation.

## Data Availability

The datasets presented in this study can be found in online repositories. The names of the repository/repositories and accession number(s) can be found in the article/Supplementary material.
