# Supplementary material for: Explainable machine learning for 2-year functional outcomes in older adults with aneurysmal subarachnoid hemorrhage in North China: external validation and health-system context
Source: Front Neurol. 2026 Sep 25;17:1955260. doi: 10.3389/fneur.2026.1955260 (PMC13649464; doi:10.3389/fneur.2026.1955260)
Supplement: Supplementary file 1 [file Supplementary_file_1.DOCX]

**Supplementary Material**

1. **Supplementary Figure 1. LASSO logistic regression for variable selection**
2. **Supplementary Figure 2. Confusion matrices of the LASSO logistic regression model across training, internal validation, and external validation sets**
3. **Supplementary Figure 3. Pairwise comparison of model discrimination using DeLong's test.**
4. **Supplementary Figure 4. SHAP interaction analysis of age and neurological severity in the GBM model.**
5. **Supplementary Figure 5. Boruta all-relevant feature selection versus LASSO in the development cohort.**
6. **Table S1 SHAP interaction analysis: age × Hunt–Hess grade, by age–severity stratum.**
7. **Table S2. Neurosurgery department capacity at participating hospitals.**

**Supplementary Figure 1. LASSO logistic regression for variable selection.**

(A) Ten-fold cross-validation curve for the LASSO logistic regression model, with binomial deviance plotted against log(λ). Each red dot represents the mean cross-validation error at a given λ value, with error bars indicating ±1 standard error. The left dashed vertical line marks λ.min (the value minimizing cross-validation error, retaining 14 variables), and the right dashed vertical line marks λ.1se (the largest λ within one standard error of the minimum, retaining 9 variables).
(B) LASSO coefficient shrinkage paths for all candidate variables. Each colored line represents one variable, showing how its coefficient is shrunk toward zero as the penalty parameter λ increases (log scale). Numbers along the top axis indicate the number of non-zero coefficients at each λ value.

**Supplementary Figure 2. Confusion matrices of the LASSO logistic regression model across training, internal validation, and external validation sets.**

Confusion matrices showing the classification performance of the model in (A) the training set (n = 640), (B) the internal validation set (n = 274), and (C) the external validation set (n = 499). Each matrix displays the proportion (%) and absolute number of true positives (Prediction Yes / Target Yes), false positives (Prediction Yes / Target No), false negatives (Prediction No / Target Yes), and true negatives (Prediction No / Target No).

**Supplementary Figure 3.** Pairwise comparison of model discrimination using DeLong's test.

Heatmap displaying pairwise p-values from DeLong's test comparing the area under the receiver operating characteristic curve (AUC) among ten machine learning models: Logistic Regression, Support Vector Machine (SVM), Gradient Boosting Machine (GBM), Neural Network, Random Forest, XGBoost, K-Nearest Neighbors (KNN), AdaBoost, LightGBM, and CatBoost. Colour intensity represents the −log₁₀(p-value) (scale: 0–15), with darker shades indicating greater statistical significance. Significance thresholds are denoted as *p < 0.05, **p < 0.01, and ***p < 0.001. Diagonal cells (self-comparisons) are indicated by dashes (—).

**Supplementary Figure 4.** SHAP interaction analysis of age and neurological severity in the GBM model.

(A) Mean absolute SHAP interaction values for the ten largest pairwise feature interactions. (B) Age × Hunt–Hess interaction dependence plot, colored by Hunt–Hess category. (C) Distribution of the age × Hunt–Hess SHAP interaction value within four age–severity strata, with sample size and observed poor-outcome proportion for each stratum (see also Table S1).

**Supplementary Figure 5.** Boruta all-relevant feature selection versus LASSO in the development cohort.

Random-forest Gini importance (log scale) for each candidate predictor across 100 Boruta iterations, benchmarked against a maximum shadow (permuted-noise) feature. Boxes are colored by Boruta outcome (confirmed, tentative, or rejected); the dashed line marks the maximum shadow importance. Asterisks flag predictors also retained by LASSO in the development cohort. All Boruta-confirmed predictors were also selected by LASSO.

**Table S1.** SHAP interaction analysis: age × Hunt–Hess grade, by age–severity stratum

| Age – Hunt–Hess stratum | n | Poor 2-year outcome | Mean SHAP interaction value (Age × Hunt–Hess) |
| --- | --- | --- | --- |
| Younger (<70y) + Low Hunt–Hess | 701 | 20.3% | +0.068 |
| Younger (<70y) + High Hunt–Hess | 189 | 70.4% | −0.208 |
| Older (≥70y) + Low Hunt–Hess | 364 | 45.1% | −0.074 |
| Older (≥70y) + High Hunt–Hess | 159 | 90.6% | +0.170 |

A positive interaction value indicates that age and Hunt–Hess grade jointly amplify the predicted risk within that stratum; a negative value indicates a mutually offsetting effect. The concordant-severity stratum (older age with high Hunt–Hess grade) showed both the highest mean interaction value (+0.170) and the highest observed poor-outcome rate (90.6%), consistent with a synergistic amplification captured by the model. In contrast, the discordant stratum (younger age with high Hunt–Hess grade) showed a negative interaction value (−0.208), suggesting the model partially offsets the risk associated with high-grade injury in younger patients, plausibly reflecting greater physiological reserve.

## **Table S2.** Neurosurgery department capacity at participating hospitals

| **Location of**  **Hospital** | **Role** | **Neurosurgery beds (total)** | **Neurosurgery wards / NICU beds** | **Annual neurosurgery workload** |
| --- | --- | --- | --- | --- |
| **Zunhua** | Training + internal validation | Not publicly disclosed (only 3 attending physicians listed publicly) | Single general ward (department is a Tangshan municipal key developing discipline) | ~200 minimally invasive intracranial hematoma surgeries/yr (partial figure; overall department volume not disclosed) |
| **Tangshan** | Training + internal validation | 65 (Neurosurgery Ward I only; department est. 1982, Tangshan municipal key discipline) | 1 general ward + NICU 20 beds (within the 65) | >20,000 cumulative admissions for intracranial tumors/vascular malformations; >10,000 cranial surgeries (cumulative, historical) |
| **Xingtai** | Training + internal validation | 43 (largest neurosurgery bed count among prefecture-level hospitals in Hebei) | Neurosurgery Ward I (trauma subspecialty); department established 1989 | Not publicly disclosed |
| **Cangzhou** | Training + internal validation | 398 (328 general + 70 NICU) | 8 wards (Neurosurgery Depts. 1–8, incl. dedicated NICU); provincial key clinical specialty | >5,000 surgeries/yr; >20,000 outpatient/ER visits/yr; >9,000 discharges/yr |
| **Shijiazhuang** | External validation | 362 (open beds) | 7 subspecialty wards (incl. dedicated critical-care ward); national/provincial key clinical specialty (Grade A) | ~25,000 outpatient visits/yr; ~13,000 ER visits/yr; ~8,000 admissions/yr; ~5,600 surgeries/yr |

Note: Department-level data (bed numbers, ward structure, annual workload) were obtained from each hospital's official department webpage or publicly available physician-referral platforms (accessed 2026) and refer to the department's current configuration, which may differ from the configuration during the study period. Where figures were not publicly available.
